# Mapping SET1B chromatin interactions with DamID using DamMapper, a comprehensive Snakemake workflow

**DOI:** 10.1186/s12864-025-12075-x

**Published:** 2025-10-14

**Authors:** Niek Wit, James Bertlin, Antony Hynes-Allen, Jelle van den Ameele, James Nathan

**Affiliations:** 1 Cambridge Institute of Therapeutic Immunology and Infectious Disease (CITIID), Department of Medicine, University of Cambridge, Cambridge, UK; 2 Department of Clinical Neurosciences, University of Cambridge, Cambridge, UK; 3 Medical Research Council Mitochondrial Biology Unit, Cambridge, UK

**Keywords:** DamID, Hypoxia, Gene regulation, Bioinformatic workflow

## Abstract

**Background:**

DNA adenine methyltransferase identification followed by sequencing (DamID-seq) is a powerful method used to map genome-wide chromatin-protein interactions. However, the bioinformatic analysis of DamID-seq data presents significant challenges due to the inherent complexities of the data and a notable lack of comprehensive software solutions for data-processing and downstream analysis.

**Results:**

To address these challenges, we present a comprehensive bioinformatic workflow for DamID-seq data analysis, DamMapper, using the Snakemake workflow management system. Key features include straightforward processing of multiple biological replicates, visualisation of quality control, such as correlation heatmaps and principal component analysis (PCA), and robust code quality maintained through continuous integration (CI). Reproducibility is ensured across diverse computational environments, including cloud computing and high-performance computing (HPC) clusters, through the implementation of software environments (Conda) and containerisation (Docker/Apptainer). We validate this workflow using a previously published DamID-seq dataset and apply it to analyse novel datasets for proteins involved in the hypoxia response, specifically the transcription factor HIF-1α and the histone methyltransferase SET1B. This application reveals a strong concordance between our HIF-1α DamID-seq results and ChIP-seq data, and importantly, provides the first genome-wide DNA binding map for SET1B.

**Conclusions:**

This work provides a validated, reproducible, and feature-rich workflow that overcomes common hurdles in DamID-seq data analysis. By streamlining the processing and ensuring robustness, DamMapper facilitates reliable analysis and enables new biological discoveries, as demonstrated by the characterization of SET1B binding sites. The workflow is available under an MIT license at https://github.com/niekwit/damid-seq.

**Supplementary Information:**

The online version contains supplementary material available at 10.1186/s12864-025-12075-x.

## Background

**Fig. 1:**
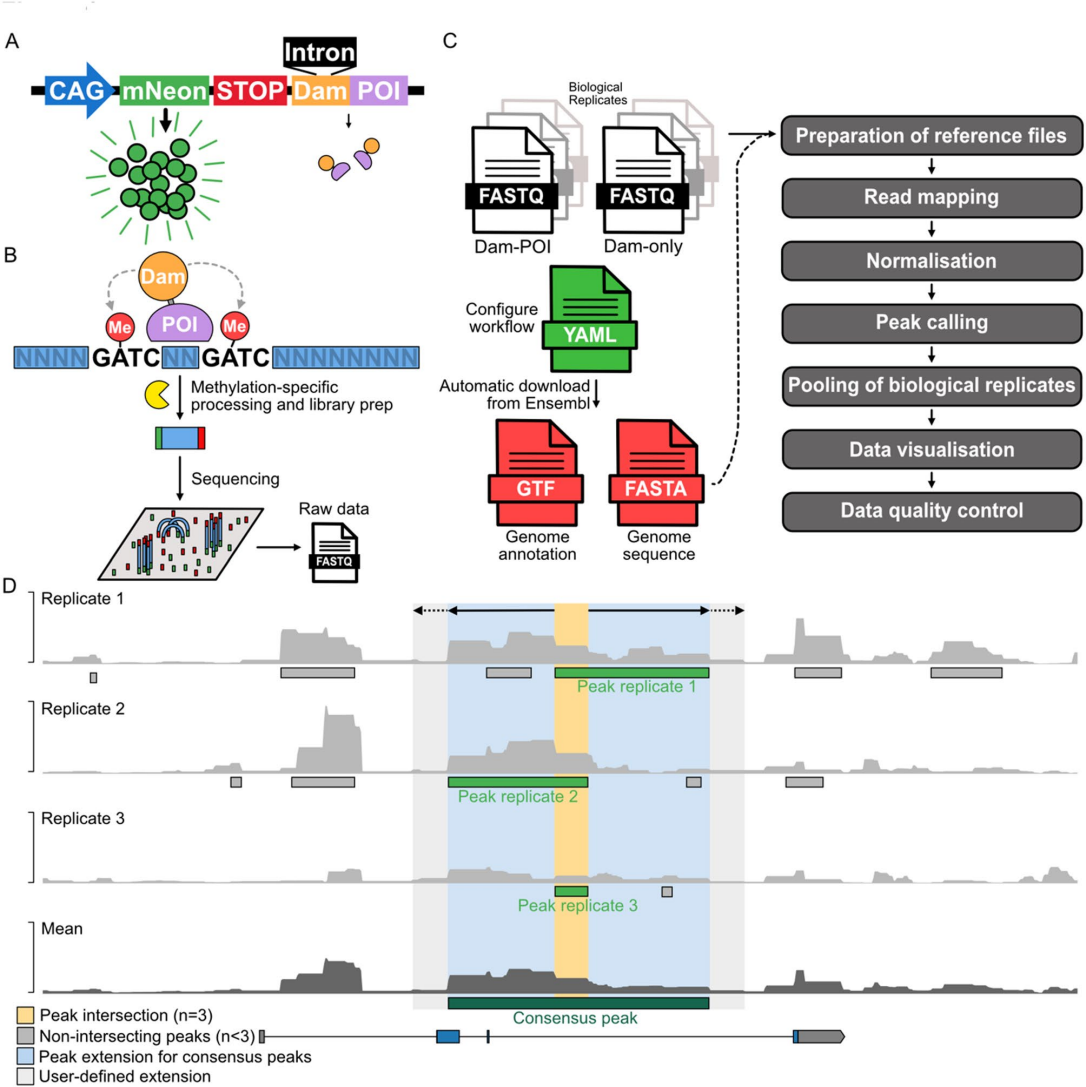
DamID and DamMapper DamID-seq Workflow. **A** Expression construct of Dam-protein of interest (POI). Transcription of a fluorescent marker gene (mNeon) is driven by a CAG promoter. Low levels of Dam-POI are ensured by reinitiation of translation of the gene after the stop codon, which happens at low levels in eukaryotes [16, 17]. The intron inside the Dam coding sequence prevents functional expression in prokaryotes, and thus unwanted methylation of the plasmid. **B** Experimental design of mapping chromatin interactions of a protein of interest (POI) using DamID-seq. When the POI binds a genomic target, the surrounding, accessible adenines in GATC motifs are methylated. Using methylation-sensitive cloning, the fragments between these modified sites are isolated and sequenced. DamMapper can then be used to map these regions and further process this data to identify regions of Dam-POI enrichment over the Dam only control. **C** DamID-seq analysis workflow. Using a YAML file, the user can configure different aspects of the analysis by DamMapper, such as which genome sequence to use, normalisation methods to apply and statistical cutoffs for peak calling. First, DamMapper downloads the relevant genome annotation (GTF) and sequence (FASTA) files from the Ensembl repositories, which are then used to prepare a Bowtie2 index to which raw sequencing data will be aligned against. There are optional steps to remove certain specified genes from the FASTA files prior to index generation or filter out reads that align to another specified sequence (user-provided in FASTA format) before genome alignment commences. After quality trimming reads are aligned to the genome index. Single-end fragments are extended to 300 bp or the nearest GATC, prior normalisation to the Dam-only control using damidseq_pipeline. To identify sites of Dam-POI enrichment over the Dam-only control, peaks are first called on individual replicates. A flexible consensus peak calling approach is then applied to identify peaks that only occur in a user-specified number of replicates, after which these peaks are annotated. To visualise the DamID data, BigWig files are generated by DamMapper, as well as plots that show peak distribution across different genomic features, among others. Finally, to help assess data quality a variety of quality control analyses are performed, such as principal component analysis of aligned fragments and correlation analysis of BigWig files. **D** Consensus peak calling strategy. Consensus peaks are determined by intersecting peaks from individual samples. The user can set the minimum number of samples to contribute to call a consensus peak. Then, the intersecting region is extended to the outermost boundaries of the individual peaks. Optionally, the consensus peaks can be further extended by a user-defined length

Mapping the genomic locations of DNA-binding proteins is essential for understanding gene regulation, chromatin organization, and other cellular processes. Chromatin immunoprecipitation followed by sequencing (ChIP-seq) is the most widely used technique for this purpose [1]. DamID-seq (DNA adenine methyltransferase identification followed by sequencing) offers an alternative to ChIP-seq [2, 3], particularly when ChIP-seq is challenging due to limitations such as a lack of suitable antibodies or low cell numbers. In DamID-seq, the DNA-binding protein of interest (POI) is fused to the *E. coli* DNA adenine methyltransferase (Dam). The Dam enzyme methylates adenines at position 6 (m6dA) in 5’-GATC-3’ sequences [4] that are accessible to the fusion protein and in spatiotemporal proximity (Fig. 1A). To identify these methylated regions, genomic DNA is isolated and treated with DpnI, which digests adenine-methylated GATC sequences. Adapters are then ligated to the ends of these methylated fragments and subsequent DpnII digestion ensures that fragments containing unmethylated GATC sequences are not amplified in the DamID PCR step. Finally, after library preparation, the DamID amplicons are sequenced using short-read Illumina sequencing [5] (Fig. 1A, B).

The low abundance of m6dA in genomes of eukaryotes [6], ensures that endogenous m6dAs do not interfere with the detection of the adenosine methylation generated by Dam. Endogenous m6dAs are also associated with a 5’-SAGGY-3’ motif [7]. Therefore, they are not processed with the DamID cloning protocol that only extracts m6dA-modified 5’-GATC-3’-flanked sequences. Dam-catalysed m6dA formation thus forms a binding history of the protein of interest (POI) [8, 9]. More recent adaptations allow DamID-seq-based profiling of a wide range of aspects of chromatin biology, including accessibility [10] and histone-modifications [11, 12], and have enabled protein-chromatin interactions to be determined within specific cell lineages in vivo [13, 14] and at single-cell resolution [11, 15] (Fig. 1B).

Analysing DamID-seq data requires a specialised toolset because of two unique aspects of the technique: (1) As Dam can non-specifically methylate accessible regions of the genome [10, 17, 18] it is essential to include a Dam-only control. By comparing the sequencing reads from a Dam-fusion protein experiment to those from Dam alone, one can infer the genuine binding sites of the POI. (2) DamID-seq operates at GATC-site resolution. Since standard normalisation methods used in ChIP-seq do not take the above aspects into account, and because the background signal in DamID-seq can significantly impact data quality, several DamID-seq-specific normalisation algorithms have been developed [19–21]. Among these, “damidseq_pipeline” [19] has been most widely adopted as a powerful and versatile pipeline, but its scope remains limited. It does not perform quality control steps and only allows processing of paired Dam-fusion/Dam-only samples thus preventing robust handling of multiple biological replicates with different numbers of Dam-fusion and Dam-only samples. Additionally, it does not account for plasmid-derived reads from transient transfection experiments [9, 22], and there is no built-in functionality for straightforward downstream analysis.

Here, we present DamMapper, a Snakemake-based workflow for DamID-seq data analysis that relies on, but also significantly expands upon the functionality of the original “damidseq_pipeline”. Furthermore, our workflow incorporates best practices for reproducible computational research [23], including:

- Biological Replicates: Seamlessly handles multiple biological replicates, improving statistical power and allowing for robust identification of binding sites.
- Extensive Quality Control: Generates a comprehensive suite of QC plots and metrics at various stages of the pipeline, enabling thorough assessment of data quality.
- Reproducibility: Supports software environments (Conda [24]) and containerisation (Apptainer, formerly Singularity [25], and Docker (https://www.docker.com)) to ensure that the analysis can be reproduced exactly on different systems and at different times. For each version of the workflow, we have uploaded a Docker image to Docker Hub (https://hub.docker.com/r/niekwit/damid-seq/). This image contains all Conda environments required to run the workflow and will be downloaded automatically upon running the workflow and converted on-the-fly to an Apptainer image by Snakemake.
- Scalability: As it is built using Snakemake [26], the workflow is inherently scalable and can be run on local machines, HPC clusters (using cluster configuration profiles for SLURM, etc.), and cloud platforms (e.g., Google Cloud, AWS) without requiring any modifications to the code base.
- Automated Reporting: Generates a comprehensive HTML report summarising the analysis, including QC plots, results tables, and parameter settings.
- Code quality and version control: Code base is version-controlled and is hosted on an open-source repository (https://github.com/niekwit/damid-seq). Moreover, for each released version Zenodo [27] mints a persistent digital object identifier, which makes the software easily citeable, while also creating an archive of the code. Finally, the repository employs CI from a Snakemake-derived template (https://github.com/snakemake-workflows/snakemake-workflow-template) via GitHub Actions to ensure code quality and stability.
- Thorough Documentation: Provides comprehensive documentation to aid in its usage and DamID-seq experimental design, hosted at https://damid-seq.readthedocs.io/.

We first validated the workflow through a proof-of-principle reanalysis of published DamID-seq data [28]. Following validation, we demonstrated its utility by analysing Hypoxia Inducible Factor-1α (HIF-1α) DamID-seq data, benchmarking it against published ChIP-seq and gene expression data (RNA-seq) [29]. The hypoxia pathway is a critical cellular adaptation mechanism to low oxygen conditions and plays a vital role in development and physiology [30, 31]. Under low oxygen conditions, the transcription factor HIF-1α evades proteasomal degradation mediated by the von Hippel-Lindau (VHL) ubiquitin ligase [32]. HIF-1α then, in a complex with HIF-1β, activates the transcription of genes involved in pathways such as glucose metabolism [32]. Dysregulation of the hypoxia pathway is implicated in tumour growth and metastasis, making it a key target for therapeutic intervention [33]. HIF-1-mediated transcription has been extensively studied, which makes it an excellent benchmark. Finally, we applied DamMapper to novel DamID-seq data for SET1B, a HIF-1-interacting histone modifying enzyme [29], providing the first comprehensive landscape of its chromatin interactions.

## Methods

### Cell culture

HeLa cells were maintained in DMEM (Sigma Aldrich) and supplemented with 10% FCS. Hypoxic cell culture was in a Whitley H35 Hypoxystation (Don Whitley Scientific) at 37 °C, 5% CO_2_, 1% O_2_ and 94% N_2_. Cells were confirmed mycoplasma negative (Lonza, MycoAltert), and authenticated by short tandem repeat profiling (Eurofins Genomics).

### Cloning

pCAG-mNeon-i4Dam was constructed by Gibson assembly into pCAG-IRES-GFP (pCIG, gift from P. Vanderhaeghen) of monomeric NeonGreen (mNeon) as an upstream open reading frame, followed by two stop codons and a frame shift (TAATAAC), and Dam from Addgene #59217 [8] with a C-terminal Myc-tag. To allow transient transfection of DamID [22] an intron was inserted into Dam by ligating oligos with a modified synthetic intron (‘IVS’) from [34] into the BamHI restriction site within Dam, between the 3rd and 4th helix of the DNA-binding domain of the Dam methylase [35].

SET1B and HIF-1α constructs derived from [29] were cloned into the pCAG-mNeon-i4Dam vector by Gibson assembly using NEBuilder HiFi (NEB E5520S) and the primers indicated in Table 1. To generate pCAG-mNeon-i4Dam-SET1B, a scaffold plasmid was first assembled in a three-piece Gibson reaction containing both SET1B PCR amplicons, before this scaffold and the original template vector were digested by FastDigest NotI (Thermo FD0594) and NotI-digested fragments substituted by T4 DNA Ligase (NEB M0202L) to ensure sequence fidelity. Plasmids used for DamID-seq experiments were amplified in dam^–^/dcm^–^ Competent E. coli (NEB C2925H).

**Table 1:** Primers used for cloning HIF-1α and SET1B into pCAG-mNeon-i4Dam

Dam-HIF-1α_Fw	TCATCTCTGAAGAGGATCTGCTCGAGAGATCTATGGAGGGCGCCGGCGGCGCGAACGACAAGAA
Dam-HIF-1α_Rv	TATCATGTCTGATGCGGCCGCTTAGATCTCAGTTAACTTGATCCAAAGCTCTGAGTAATTC
Dam-SET1B_Fw	AACTCATCTCTGAAGAGGATCTGCTCGAGAGATCTATGGAGAACAGTCACCCCCCCCACCACCACCACCA
Dam-SET1B_int-Fw	CCATTCGCCTGCCCTCCTTCAAGGTCAAGAGGAAGGAGC
Dam-SET1B_int-Rv	GCTCCTTCCTCTTGACCTTGAAGGAGGGCAGGCGAATGG
Dam-SET1B_Rv	TATCATGTCTGATGCGGCCGCTTAGATCAAGCTTCTAGTTGAGGGTCCCCCGGCAGTTCTCGGAGCC

### DamID

2 × 10^5^ HeLa cells were plated in each well of a six-well plate. After 24 h, cells were transfected with pCAG-mNeon-i4Dam, pCAG-mNeon-i4Dam-HIF-1α, or pCAG-mNeon-i4Dam-SET1B using TransIT-HeLaMONSTER (Mirus Bio MIR2900) according to the manufacturer’s instructions and immediately transferred to 1% O_2_ for a further 24 h. Cells were harvested by trypsinisation, washed in PBS, pelleted and stored at − 80 °C prior to DNA extraction. Cells were processed for DamID as described previously [5]. DamID fragments were prepared for Illumina sequencing according to a modified TruSeq protocol. Sequencing was performed as paired end 50 bp reads by the CRUK Genomics Core Sequencing facility on a NovaSeq 6000.

### Bioinformatics analysis

A Snakemake workflow was employed for DamID analysis (https://github.com/niekwit/damid-seq [36]),. Briefly, quality trimming was performed using Trim Galore [37] to remove adapter sequences. Pre and post trimming FastQC/MultiQC (https://github.com/s-andrews/FastQC [38]) analysis was performed to assess read/trimming quality. Read mapping to the human reference genome (Ensembl GRCh38, build 110) was performed using Bowtie2 [39]. Normalisation to the Dam-only control was carried out using ”damidseq_pipeline” [19] with BAM files as input. If the sequencing data consisted of single-end reads, extension to 300 bp or the nearest GATC was carried out, prior to Dam-only normalisation using a custom Perl script, which was based on an identical functionality from ”damidseq_pipeline”. This process aims to reconstruct the original fragment size. The resulting bedGraph files were then quantile normalised using a custom Python script, which transforms the input data such that the distributions of scores across all samples become identical, achieved by ranking values within each sample and replacing them with the average of the corresponding ranks across all samples. Scores from all bedGraph files are organised by shared genomic coordinates and normalised across the samples. This quantile normalisation step is optional and can be applied to correct for potential technical variation, such as batch effects. Mean BigWig files from similar conditions were generated using bedGraphToBigWig [40], wiggletools [41] and wigToBigWig [40]. The log transformation of the bedGraph files was reversed, and the midline changed to 1, only for visualisation of signal using pyGenomeTracks [42]. Peak calling was performed using MACS on individual replicates first [43]. Consensus peaks were then determined as follows: first, using BEDTools multiint [44], common intervals among all replicates, and subsets thereof, were identified. Second, using a custom Python script, the intervals that were derived from a user-defined number of replicates were retained. These peaks were subsequently extended to the left-most and right-most boundaries of the individual peaks that contributed to a given consensus peak (Fig. 1C). Finally, an optional further peak extension can be applied. Consensus peaks were annotated with the R package ChIPseeker [45].

Profile plots and heatmaps were generated with the deepTools [46] subcommands computeMatrix, and plotProfile and plotHeatmap, respectively. Venn diagrams were made using the Eulerr R package [47]. Statistical significance of overlap in Venn diagrams was performed using either BEDTools fisher (single comparison) [44] or Genomic Association Test [48]. Motif analysis was carried out with HOMER [49]. Finally, g:Profiler2 [50] was used to carry out functional gene set enrichment.

### Runtime statistics for dammapper

For both the SOX9 and HIF-1α/SET1B experiments, using the report functionality from Snakemake, we generated a JSON file that contains runtime statistics for each rule in the workflow. These can be found summarised in Additional file 1: Table S1A, B together with the allocated CPU count per rule. Both analyses were run on a Dell Precision 7820 system with a dual CPU (2x Intel Xeon Silver 4214, 12 CPU cores/24 threads in parallel per CPU, at 3.200 GHz) With 128 GiB of RAM, running Ubuntu GNU/Linux (20.04.6 LTS x86_64). When 40 threads were allocated to the entire analysis, the total runtime was 3 h, 6 min, 22 s, and 5 h, 47 min, 11 s, for the SOX9 and HIF-1α/SET1B data sets, respectively.

## Results

### DamMapper addresses existing challenges with DamID-seq data analysis

The DamMapper workflow is designed to address DamID-specific data analysis challenges and extend the workflow beyond the functionality of existing methodology (i.e. damidseq_pipeline).

A specific limitation of damidseq_pipeline was the normalisation of DamID data from Dam-fusion protein samples against corresponding Dam-only controls, thus constraining by a one-to-one pairing requirement. To allow experimental designs in which the numbers of Dam-fusion and Dam-only samples are unequal, the DamMapper workflow implements a combinatorial pairing approach [22]. It generates all possible pairings of Dam-fusion and Dam-only samples, with symbolic links to the raw data created in separate directories for each pair. This structure permits the execution of data normalisation with damidseq_pipeline on all possible pairings, thereby fully utilising all available data, while also increasing the statistical power as more replicates become available for consensus peak calling.

A second challenge for DamID-seq analysis relates to GATC density. This impacts DamID-seq signals in several ways: (1) regions without specific methylation can generate a large degree of noise in the final ratio file. To mitigate this, the damidseq_pipeline normalisation algorithm divides the read counts into GATC fragments and then excludes fragments that lack reads [19], and (2) single-end sequencing data may not fully reflect the actual fragment that was sequenced. To reconstruct the original fragment size an extension is performed to 300 bp or the nearest GATC site.

Finally, a potential artifact in DamID can arise when the Dam fusion protein or Dam-only control binds to its own accessible expression construct (for example the transiently transfected plasmid or the integrated viral vector), thereby generating excessive amounts of reads. This can happen in bacteria when cloning the construct, or at a later stage in eukaryotes. To prevent the Dam-mediated methylation of the expression construct in bacteria, an intron is placed inside the Dam coding sequence which prevents functional expression of Dam in prokaryotes (Fig. 1B) [22]. To prevent binding artifacts from self-targeting in eukaryotes, we implemented two complementary strategies that can exclude this signal from the analysis:

1. Masking the sequence of the investigated gene (either the entire gene sequence or only exonic regions) within the reference genome FASTA file used for Bowtie2 alignment. This prevents reads originating from the Dam fusion construct from mapping to the corresponding genomic locus.
2. Performing a pre-alignment step against a FASTA file containing only the Dam-fusion or complete plasmid sequence. Only reads that fail to align to the fusion construct are retained for subsequent mapping to the full reference genome.

### DamMapper workflow description

The DamMapper workflow implements Snakemake, a widely used workflow management system [26]. Snakemake allows for the definition of complex workflows in a concise and readable manner, automatically handles dependencies between steps, and facilitates parallel execution. The workflow (Fig. 1C) consists of the following key stages:

1. Preparation of reference files: a FASTA file where the region(s) of the Dam fusion gene(s) can be masked is used to generate the Bowtie2 index, and a file that maps GATC sites is generated with a Python script that employs multithreading.
2. Read Mapping: Quality trimmed sequencing reads (FASTQ files) are mapped to the reference genome using Bowtie2 [39]. Single-end fragments are extended at the 3’ end to 300 bp or the nearest GATC site.
3. Normalisation: each biological replicate is normalised to a corresponding Dam-only control and corrected for GATC density using “damidseq_pipeline” [19]. An optional quantile normalisation can be applied at this step.
4. Peak calling: MACS [43]or a peak calling algorithm associated with “damidseq_pipeline” (https://github.com/owenjm/find_peaks) can be used to call peaks on individual replicate samples.
5. Pooling of biological replicates: to facilitate data visualisation, mean BigWig files are generated by averaging signal across biological replicates within each condition. Consensus peaks are determined by identifying regions of overlap among peaks called from individual samples. Crucially, each consensus peak is annotated with the number of samples supporting it, allowing users to filter for peaks present in a user-defined minimum number of replicates (Fig. 1D). Finally, an optional peak extension step can be performed.
6. Data visualisation:
  - Heatmap and profile plot of the pooled data.
  - Peak annotation plots (feature distribution and distance to TSS).
7. Data quality control:
  - PCA plot of all samples (BAM files).
  - Clustered Spearman correlation heatmap of all samples (BAM files).
  - Alignment rates for all samples.
  - Fraction of reads in peaks.
  - FastQC/MultiQC analysis of raw and quality trimmed FASTQ files.

We further optimised the workflow by:

1. Replacing the original Perl GATC track maker with a faster, multithreaded Python version.
2. Enabling parallel alignment of individual trimmed FASTQ files via Snakemake.
3. Implementing a separate script for single-end read extension that can be run in parallel by Snakemake, previously performed serially within “damidseq_pipeline”.

Finally, a single YAML file controls all workflow parameters and computational resources described above, which provides flexibility and allows users to pass additional arguments to individual tools used in the workflow to tailor the analysis to their specific needs.

### Proof-of-principle re-analysis of published DamID data

**Fig. 2:**
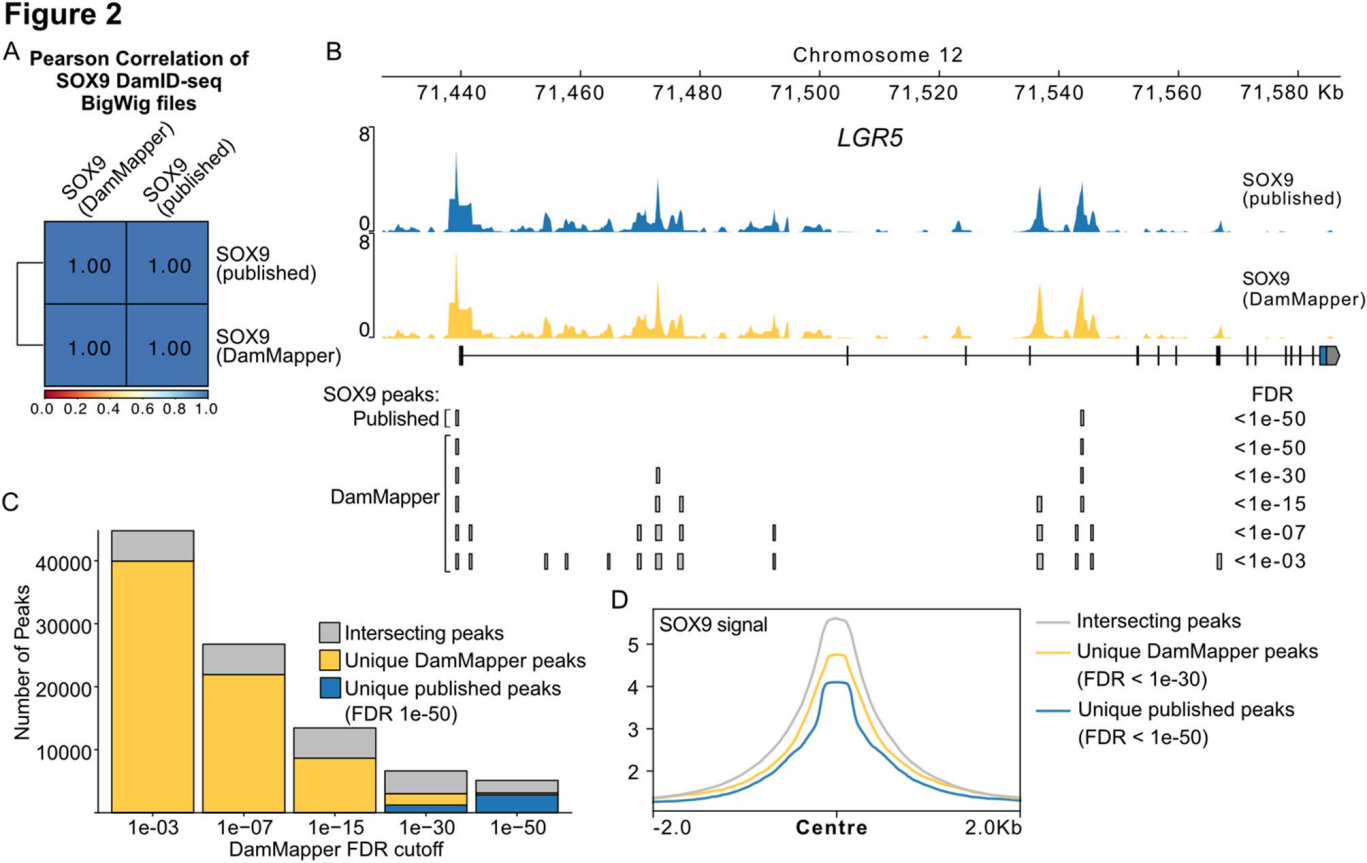
Validation of DamMapper with a Published DamID-seq Dataset. **A** Pearson correlation analysis of SOX9 DamID BigWig files. Generated with multiBigwigSummary and plotCorrelation from deepTools. **B** SOX9 DamID signal and peaks plotted using pyGenomeTracks at the *LGR5* locus. Peak sets generated with various FDR cutoffs for DamMapper are shown. Published peaks were called using MACS2, while DamMapper employs MACS3.**C** Bar graph showing number of peaks in the intersection group and unique peaks, between the published and DamMapper-generated SOX9 peak sets

To validate our workflow, we first reanalysed published DamID-seq data mapping SOX9 chromatin interactions in human fetal lung tip progenitors [28]. We first compared merged BigWig files from the original study (GSE189885) with those generated by DamMapper using deepTools multiBigwigSummary and plotCorrelation. The reanalysed data showed a near-perfect Pearson correlation with the published results (Fig. 2A).

We then investigated the binding patterns at a well-established SOX9-bound locus and at a genome-wide level. At the *LGR5* locus, SOX9 peaks were identical between MACS2 and DamMapper when using an FDR cutoff of 1e-50. While some regions (e.g., exon 4 signal) were initially missed by both, this was due to the stringent cutoffs as additional peaks were called with more lenient settings (Fig. 2B). Genome-wide, DamMapper captured all published peaks from MACS2 using an FDR cutoff of 1e-15 (Fig. 2C), while also detecting additional peaks. These differences most likely stem from (1) algorithmic differences in peak calling between MACS2 and MACS3 (Fig. 2C, D), and (2) different filtering approaches between the published analysis and DamMapper, as the original analysis ran MACS2 With an FDR cutoff of 0.1, followed by selecting peaks with FDR < 1e-50, while DamMapper runs MACS3 with an FDR cutoff 1e-50 directly.

### Mapping of HIF-1α chromatin interactions using DamID-seq and comparison with ChIP-seq data

Having validated our workflow on published data, we wanted to further validate it with a novel DamID-seq dataset and compare it to published ChIP-seq data. To this end, we utilised HIF-1α as a well-established benchmark to further validate DamMapper, given its extensive genomic binding profile characterised by previous ChIP-seq studies [29, 51, 52]. We chose a 24-hour exposure to 1% oxygen after transient Dam-HIF-1α transfection in HeLa cells to ensure adequate expression of the fusion protein and to capture a comprehensive view of HIF-1α binding dynamics, including both early and late interactions.

**Fig. 3:**
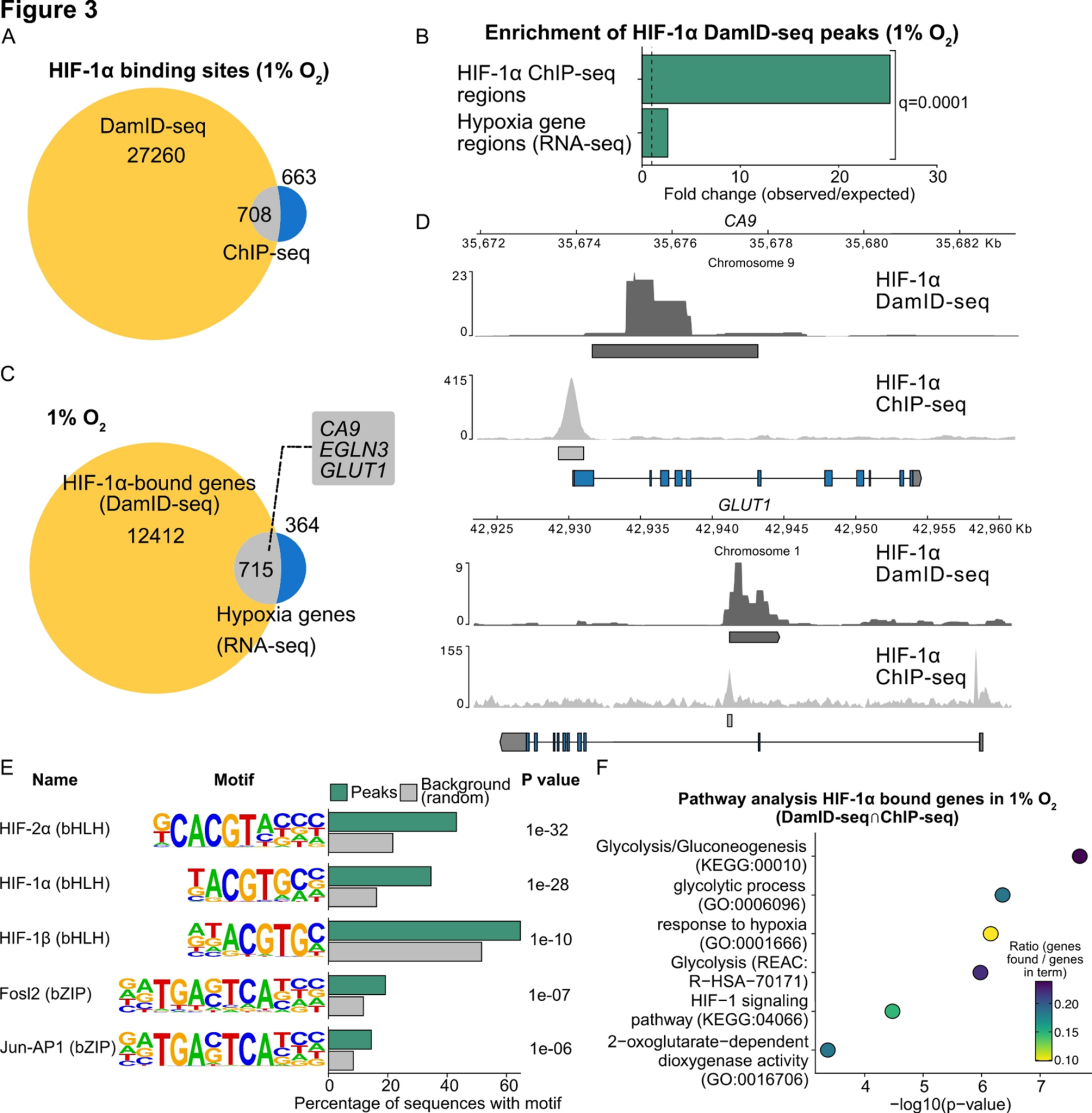
Comparison of HIF-1α DamID-seq and ChIP-seq. **A** Venn diagram of HIF-1α binding sites identified by DamID and ChIP-seq. **B** Quantification of spatial relationship of Venn diagrams in Fig. 3 A,C (as quantified with Genome Association Tester, n=10,000). **C** Venn diagram of HIF-1α-bound genes (DamID) and hypoxia-upregulated genes (RNA-seq). Hypoxia upregulated genes are defined as genes with increased transcription in 1% O_2_ for 12 hours versus 21% O_2_. **D** Example of genes bound by HIF-1α (Carbonic anhydrase 9, *CA9*, and Glucose Transporter 1, *GLUT1* (also known as *SLC2A1*) found with DamID and ChIP-seq using pyGenomeTracks. **E** Functional enrichment analysis of genes found in both HIF-1α DamID and ChIP-seq using g:Profiler2. **F** Motif analysis of regions found in both HIF-1α DamID-seq and ChIP-seq using HOMER

HIF-1α DamID-seq identified a total of 27,968 sites of enriched binding using MACS (Fig. 3A). Comparing our DamID-seq results (1% O₂ for 24 h) to publicly available HIF-1α ChIP-seq (1% O_2_ for 6 h) and RNA-seq data (1% O_2_ for 12 h) [29] revealed significant overlap. Specifically, 708 out of 1371 HIF-1α binding sites identified by ChIP-seq were also detected by DamID-seq (Fig. 3A). This enrichment was highly significant (q = 0.0001, Fig. 3B). Importantly, DamID-seq identified binding sites near well-validated HIF-upregulated genes, including *CA9*, *EGLN3*, and *GLUT1* (Fig. 3C, D), as previously determined by RNA-seq [29] (upregulated genes in hypoxia versus normoxia). This enrichment was also highly significant (q = 0.0001, Fig. 3B). It is important to note that peaks identified with DamID-seq may appear broader than their ChIP-seq equivalents, as the resolution of DamID-seq is determined by the density of GATC sequences in the genome (Fig. 3D). Motif analysis using HOMER [49] showed enrichment of HIF binding motifs at shared DamID and ChIP-seq sites (DamID-seq$\:\cap\:$ChIP-seq) (Fig. 3E). Moreover, we also observed enrichment of Jun-AP1 motifs, which were also detected in HIF-1α ChIP-seq data [51]. Genes associated with DamID-seq$\:\cap\:$ChIP-seq sites were enriched in well-established HIF target pathways, such as glycolysis (g: Profiler [50]) (Fig. 3E). In summary, these findings demonstrate the robustness of DamMapper for identifying HIF-1α binding sites.

While we found that HIF-1α DamID-seq peaks were highly enriched in ChIP-seq peaks, DamID identified a much greater number of HIF-1α-bound regions not found by ChIP-seq. This is likely explained by fundamental differences between the two techniques: ChIP-seq creates a snapshot of protein-DNA interactions at the moment of crosslinking, while DamID-seq records binding events over time. Additionally, DamID-seq may also capture proximity events between the POI and its genomic target, secondary sites resulting from chromatin looping [53] or transient binding. In conclusion, DamID-seq is capable of mapping both robust chromatin interactions and weaker binding events that may be transient or distant, which ChIP-seq would not detect.

### Chromatin interaction mapping of SET1B

Next, we wanted to map chromatin interactions of proteins known to regulate transcription but where ChIP-seq has not been possible due to the lack of suitable antibodies. We chose to examine chromatin binding of the histone methyltransferase SET domain containing 1B (SET1B), which we recently demonstrated to boost HIF target gene expression by enhancing H3K4me3 levels at HIF-target loci [29]. SET1B is a member of the COMPASS family, which also includes SET1A and MLL1-4 [54]. Although COMPASS family members have been the subject of considerable research, genome-wide binding data for SET1B, required for a complete understanding of its regulatory role, are not yet available.

**Fig. 4:**
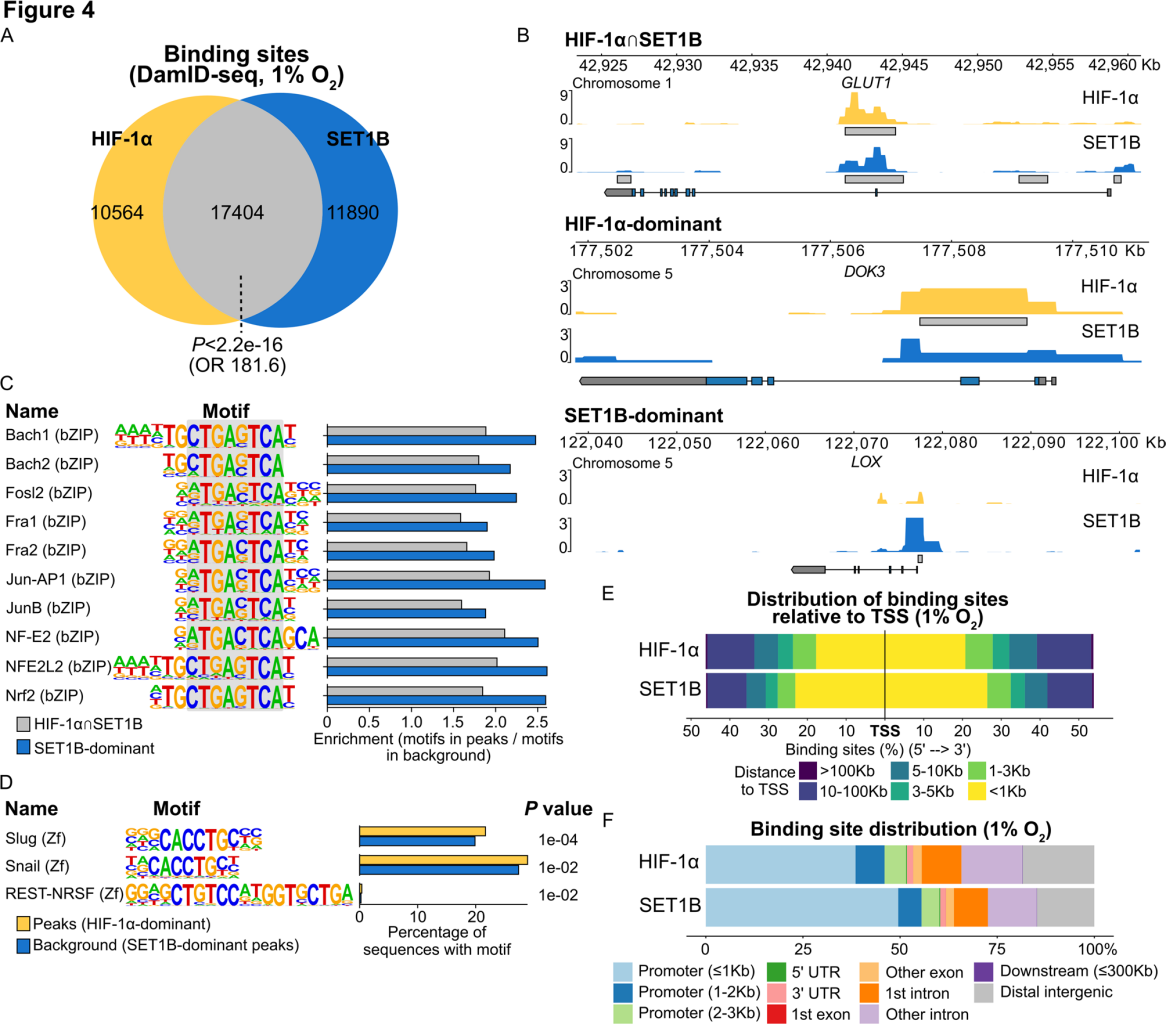
Analysis of SET1B Chromatin Mapping with DamID-seq. **A** Venn diagram of HIF-1α and SET1B bound regions as found by DamID-seq. The significance on the number of overlaps was determined with BEDTools fisher. **B** Example tracks of genes bound by HIF-1α and SET1B at 1% O_2_ using pyGenomeTracks. **C** Motif analysis of regions bound by both HIF-1α and SET1B (HIF-1α∩SET1B, random regions as background) or enriched by SET1B only (HIF-1α-dominant regions as background), using HOMER. Data shown has *P* ≤ 1e-33. **D** Motif analysis of HIF-1α dominant regions (SET1B dominant regions as background) using HOMER. **E** Analysis of distribution of all HIF-1α or SET1B binding sites relative to TSS of closest gene using ChIPseeker. **F** Analysis of HIF-1α or SET1B binding sites in different gene features using ChIPseeker

We performed SET1B DamID-seq using the same conditions as our previous HIF-1α experiments and, using DamMapper, found 29,294 unique chromatin interaction sites. These sites overlapped significantly (59.4%, *P* < 2.2e-16, OR 181.6) with HIF-1α DamID binding sites, even though we also found sites that were dominantly enriched by either HIF-1α or SET1B (Fig. 4A, B). We defined these dominant sites as genomic regions exhibiting a significant peak for one protein while the other protein’s signal, though potentially present, did not reach the threshold for peak calling (examples in Fig. 4B, middle and lower panel). To better understand SET1B binding, we first analysed motifs enriched at two categories of SET1B sites: SET1B-dominant sites and sites occupied by both SET1B and HIF-1α (Fig. 4C). This revealed that they were similarly enriched in motifs recognised by Basic Leucine Zipper Domain (bZIP) transcription factors. Notably, a common core sequence, 5’-MTGASTCA-3’, was identified across these top-ranking motifs (Fig. 4C). Interestingly, sites where HIF-1α binding was dominant did not show enrichment of these motifs relative to the SET1B-dominant binding sites that were used as background (Fig. 4D). Taken together, our motif analysis suggests that SET1B chromatin binding in low oxygen conditions, irrespective of HIF-1α presence, may also require a bZIP transcription factor. Further characterisation showed that both HIF-1α and SET1B predominantly bind near gene promoters (within 5 kb of the TSS; Fig. 4E, F), consistent with their roles in stimulating transcription. However, about 20% of binding sites were promoter-distant. These promoter-distant sites were also observed in previously published HIF-1α ChIP-seq data [51] and may represent binding to enhancer regions.

**Fig. 5:**
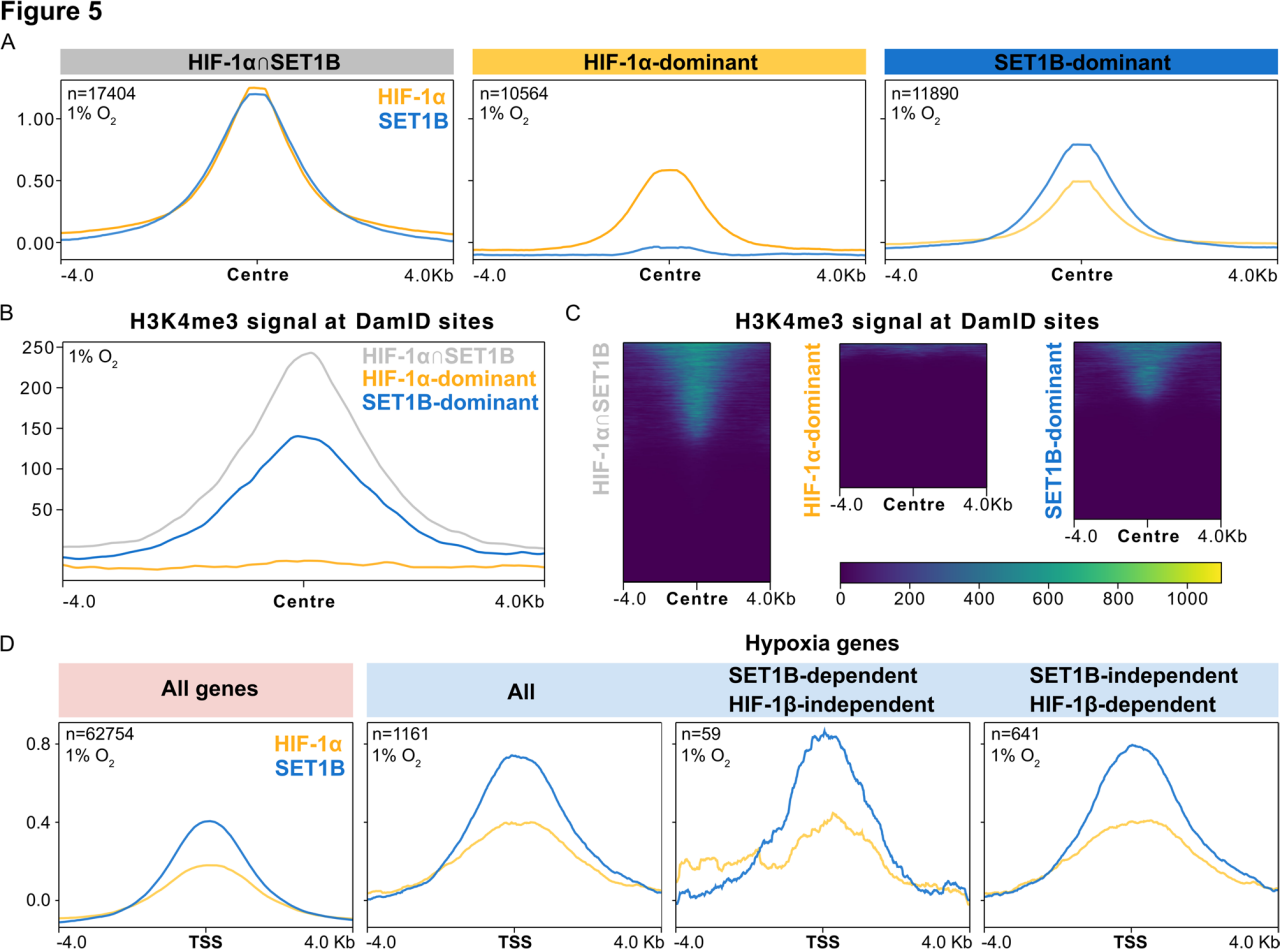
Co-localization and Genomic Profiles of HIF-1α and SET1B. **A** Profile plots of HIF-1α and SET1B scores at HIF-1α∩SET1B sites or where only either HIF-1α or SET1B were enriched using computeMatrix and plotProfile (deepTools). **B** Profile plot of H3K4me3 signal at HIF-1α and SET1B sites using computeMatrix and plotProfile (deepTools). **C** Heatmap of H3K4me3 signal at HIF-1α and SET1B sites using computeMatrix and plotHeatmap (deepTools). **D** Profile plots of HIF-1α and SET1B scores at all genes, hypoxia genes (1% O_2_, 12 hours), SET1B-dependent/HIF-1β-independent genes or SET1B-independent/HIF-1β-dependent genes using computeMatrix and plotProfile (deepTools)

At sites where peaks of HIF-1α and SET1B intersected (HIF-1α$\:\cap\:$SET1B), their occupancy levels were nearly identical (Fig. 5A). In contrast, HIF-1α-dominant regions showed very low SET1B levels, while SET1B-dominant regions still retained HIF-1α signal. This pattern indicates that while most SET1B binding occurs jointly with HIF-1α, HIF-1α chromatin binding can occur in the relative absence of SET1B.

To understand the interplay between HIF-1α/SET1B chromatin binding and the epigenetic state, we investigated H3K4me3 deposition under hypoxia (6 h, 1% O_2_, previously published data [29]). This chromatin mark is induced by hypoxia [55, 56] and its spreading is SET1B-dependent [29]. We examined H3K4me3 levels at HIF-1α- or SET1B-dominant sites as identified via DamID-seq. Notably, H3K4me3 levels were highest at sites co-occupied by HIF-1α and SET1B (Fig. 5B, C). SET1B-dominant sites also exhibited H3K4me3, albeit at lower levels, while HIF-1α-dominant sites showed almost no H3K4me3 deposition. These findings suggest that in low oxygen conditions H3K4me3 deposition may require the co-occurrence of HIF-1α and SET1B.

Finally, we examined binding of HIF-1α and SET1B at hypoxia-upregulated genes (1% O_2_ exposure for 12 h [29]). Given our previous work demonstrating that certain subsets of these differentially expressed genes rely on HIF-1 or SET1B for transcription in hypoxia [29], we explored whether there was corresponding enrichment of either HIF-1α or SET1B (Fig. 5D) on these genes. However, while an overall enrichment of both factors at hypoxia genes relative to the genome average was observed, the binding of HIF-1α and SET1B clearly segregated with the observed effects on transcriptional regulation. This suggests either (1) a more complex regulatory landscape, where HIF-1α or SET1B binding may be accompanied by an additional factor to stimulate HIF-mediated transcription, or (2) DamID-seq is detecting transient binding events by HIF-1α and SET1B, which by themselves are insufficient for transcriptional regulation.

## Discussion and conclusions

We present DamMapper, a comprehensive and reproducible Snakemake-based workflow for DamID-seq data analysis, addressing key challenges in the field. By building on the existing “damidseq_pipeline” and extending its functionality, the workflow enables streamlined processing of biological replicates, extensive quality control, and robust code quality assurance. The implementation of software environments and containerisation ensures reproducibility across diverse computational infrastructures without requiring any modifications to the code base.

While its combinatorial pairing approach enhances flexibility and statistical power by allowing unequal numbers of Dam-fusion and Dam-only samples, it may introduce noise from batch effects due to potentially “mismatched” Dam-only normalization. Additionally, the GATC extension step, used to reconstruct single-end fragment size, may introduce biases as signal in GATC-sparse regions may be spread over a wider genomic area, while in GATC-rich regions the signal may be confined to a smaller region, thereby increasing the resolution.

Our validation on both published and novel datasets demonstrates the validity of the workflow. The concordance between HIF-1α DamID and ChIP-seq data further supports the reliability of DamID-seq as an alternative or complementary method to ChIP-seq. Compared to ChIP-seq, DamID-seq intriguingly detected a far larger number of HIF-1α binding sites; more even than the number of hypoxia-responsive genes, which we also observed previously using DamID-seq for HIF-1α in human fetal lung epithelial progenitors [57]. It is possible that many of the additional HIF-1α binding sites identified via DamID-seq correspond to transient binding events or more distant interactions. Alternatively, it may indicate that HIF-1α binding alone does not guarantee gene activation and that there may be a requirement for an additional factor to initiate hypoxia-inducible transcription.

We provide the first genome-wide chromatin mapping of the histone methyltransferase SET1B in low O_2_ conditions, a critical factor for efficient activation of HIF transcriptional activity [29]. This revealed that SET1B has similar binding patterns compared to HIF-1α, and that these sites are significantly enriched in sequence motifs relating to bZIP transcription factor binding sites, mainly the AP-1 immediate-early transcription factor. Given that AP-1 (a Jun/Fos heterodimer [58]) is induced by hypoxia or by VHL-deficiency in clear cell renal carcinoma [59, 60] and its early induction is HIF-1α-independent [61], future studies could explore the potential interaction between SET1B and AP-1 under hypoxia, which may be an early and HIF1-independent response mechanism.

## Supplementary Information

Additional file 1. Runtimes and CPU usage for SOX9 analysis (Table S1A) and SET1B/HIF-1α analysis (Table S1B) DamMapper rules.

## Data Availability

The DamID datasets generated during the current study are available in the NCBI Gene Expression Omnibus repository: GSE296995. Published H3K4me3 ChIP-seq data was obtained from GSE159128 [29].
